# Herbert Stanley Ballance

**Published:** 1921-09

**Authors:** 


### HERBERT STANLEY BALLANCE, M.D., B.S. Lond.

To the great regret of his many friends and devoted patients Herbert Stanley Ballance died suddenly on June 11th, aged 56.

Although he had been confined to his bed for some time it was hoped he would make a good recovery, and the sudden fatal termination was quite unexpected.

He was educated at Mill Hill School and King's College Hospital, and took the diploma of M.R.C.S., L.R.C.P. in 1890, and in 1892 graduated M.B., B.S. London, taking his M.D. in 1894. He held the posts of Resident Medical Officer at the Brompton Hospital for Consumption ; House Physician to the General Lying-in Hospital, York Road ; and House Surgeon to King's College Hospital under Lord Lister (then Sir Joseph Lister). In 1895 he started in general practice in Weston-super-Mare.

Ballance was on the staff of the Weston-super-Mare Hospital as Surgeon and also Surgeon in charge of the X-Ray Department. The initiation of this department was entirely due to his efforts in collecting a large sum of money for the purpose, and he devoted much of his time to this work, giving to it the same painstaking care which was characteristic of all his work.

As a doctor he was gentle and considerate, and his patients regarded him with confidence and affection.

His colleague, Dr. Crouch, writes :—

" I knew Ballance intimately for over twenty-five years and saw a great deal of him, as we lived almost next door to each other.

" He was a most sincere friend, and one could always be sure that if you wanted help in any way he would put his whole heart and mind into your difficulties, and give you good advice and assistance. Apart from his work, what he really loved was motor cars, and however tired he might be would always brighten up and come to one's aid for ' magneto trouble ' or other difficulties. His knowledge of machinery was greatly helped by his perseverance and endless patience, and there is no doubt that some of his happiest hours were spent in an old suit at the garage, and there I like to think of him."

To his widow and son we tender sincere sympathy in their bereavement.

